# Elucidating the Underlying Functional Mechanisms of Breast Cancer Susceptibility Through Post-GWAS Analyses

**DOI:** 10.3389/fgene.2018.00280

**Published:** 2018-08-02

**Authors:** Mahdi Rivandi, John W. M. Martens, Antoinette Hollestelle

**Affiliations:** ^1^Department of Medical Oncology, Erasmus MC Cancer Institute, Rotterdam, Netherlands; ^2^Department of Modern Sciences and Technologies, School of Medicine, Mashhad University of Medical Sciences, Mashhad, Iran; ^3^Cancer Genomics Centre, Utrecht, Netherlands

**Keywords:** breast cancer, susceptibility loci, post-GWAS analysis, fine-scale mapping, functional analysis

## Abstract

Genome-wide association studies (GWAS) have identified more than 170 single nucleotide polymorphisms (SNPs) associated with the susceptibility to breast cancer. Together, these SNPs explain 18% of the familial relative risk, which is estimated to be nearly half of the total familial breast cancer risk that is collectively explained by low-risk susceptibility alleles. An important aspect of this success has been the access to large sample sizes through collaborative efforts within the Breast Cancer Association Consortium (BCAC), but also collaborations between cancer association consortia. Despite these achievements, however, understanding of each variant's underlying mechanism and how these SNPs predispose women to breast cancer remains limited and represents a major challenge in the field, particularly since the vast majority of the GWAS-identified SNPs are located in non-coding regions of the genome and are merely tags for the causal variants. In recent years, fine-scale mapping studies followed by functional evaluation of putative causal variants have begun to elucidate the biological function of several GWAS-identified variants. In this review, we discuss the findings and lessons learned from these post-GWAS analyses of 22 risk loci. Identifying the true causal variants underlying breast cancer susceptibility and their function not only provides better estimates of the explained familial relative risk thereby improving polygenetic risk scores (PRSs), it also increases our understanding of the biological mechanisms responsible for causing susceptibility to breast cancer. This will facilitate the identification of further breast cancer risk alleles and the development of preventive medicine for those women at increased risk for developing the disease.

## Introduction

Breast cancer, the second deadliest cancer among women worldwide, is still the most frequently diagnosed malignancy among females (Fitzmaurice et al., 2017). Different risk factors, related to the development of breast cancer, have been identified with genetic predisposition playing a pivotal role. About 10–15% of the women who develop breast cancer have a familial background of the disease and several genes have been identified that increase breast cancer risk when mutated in the germline (Collaborative Group on Hormonal Factors in Breast Cancer, 2001; Stratton and Rahman, 2008; Hollestelle et al., 2010b). Moreover, a large amount of non-coding germline variants have been identified that not only contribute to the breast cancer risk observed in individuals with a familial background, but also significantly in the general population (Lilyquist et al., 2018).

Currently identified breast cancer susceptibility genes and alleles can be stratified by their conferred risk in high, moderate and low-penetrant categories. *BRCA1* and *BRCA2* are the two most commonly mutated high-penetrance genes and about 15–20% of the familial breast cancer risk is attributable to germline mutations in one of these two genes (Miki et al., 1994; Wooster et al., 1995; Stratton and Rahman, 2008). Although germline mutations in *PTEN, TP53, STK11*, and *CDH1* also confer a high breast cancer risk, they are very rare and mostly found within the context of the cancer syndromes they cause. Hence, mutations in these genes explain no more than 1% of the familial breast cancer risk (Stratton and Rahman, 2008). A more intermediate risk of developing breast cancer is conferred by germline mutations in the genes *CHEK2, ATM, PALB2*, and *NBS1*, which are, in the general population, more prevalent than mutations in the high risk breast cancer genes. Together they explain another 5% of the familial breast cancer risk (Meijers-Heijboer et al., 2002; Vahteristo et al., 2002; Renwick et al., 2006; Steffen et al., 2006; Rahman et al., 2007; Hollestelle et al., 2010b). Interestingly, all high and moderate-risk genes identified so far have been implicated in the DNA damage response pathway (Hollestelle et al., 2010b).

Lastly, more than 170 low penetrant breast cancer susceptibility alleles have been identified through large-scale GWAS, which explain about 18% of the familial breast cancer risk (Michailidou et al., 2017). The vast majority of these GWAS-identified SNPs are, however, located outside coding regions (www.genome.gov/gwastudies). It is therefore not immediately obvious how these SNPs confer an increased risk to develop breast cancer. Moreover, since a GWAS design takes advantage of the linkage disequilibrium (LD) structure of the human genome and thus includes only SNPs tagging a particular locus, GWAS-identified SNPs usually do not represent the causal risk variants. Post-GWAS analyses are therefore imperative to identify the underlying causal SNP(s) and discern their mechanism of action. Since these causal SNPs are expected to display a stronger association with breast cancer risk than the original GWAS-identified SNPs (Spencer et al., 2011), their identification not only improves our estimates of the explained familial breast cancer risk by these SNPs, it also improves PRSs that aid in the identification of women at risk to develop breast cancer. In this review, we summarize the findings from post-GWAS analyses to date and discuss lessons learned with respect to design of these studies and the results that they have produced.

## GWAS-identified SNPs

Since 2007, when one of the first large GWASs for breast cancer was published, multiple GWASs have been performed in order to identify those SNPs associated with the development of breast cancer (Easton et al., 2007; Hunter et al., 2007; Stacey et al., 2007, 2008; Gold et al., 2008; Ahmed et al., 2009; Thomas et al., 2009; Zheng et al., 2009; Turnbull et al., 2010; Cai et al., 2011a, 2014; Fletcher et al., 2011; Haiman et al., 2011; Ghoussaini et al., 2012; Kim et al., 2012; Long et al., 2012; Siddiq et al., 2012; Garcia-Closas et al., 2013; Michailidou et al., 2013, 2015, 2017; Purrington et al., 2014; Couch et al., 2016; Han et al., 2016; Milne et al., 2017). To date, 172 SNPs have been identified that associate with breast cancer risk. One of the major driving forces behind this success is the establishment of large international research consortia such as BCAC, which facilitated large sample sizes for breast cancer GWAS. Additionally, the cooperation between different large association consortia for breast, ovarian, prostate, lung and colon cancer (i.e., BCAC, CIMBA, OCAC, PRACTICAL, GAME-ON), which led to the development of the iCOGS array and the OncoArray has also been critical. In this respect, the iCOGS array facilitated the identification of 41 and 15 new breast cancer susceptibility loci, while the latest OncoArray facilitated identification of another 65 (Michailidou et al., 2013, 2015, 2017). Although the latest GWAS on the OncoArray has identified the most novel risk loci to date, the GWAS-identified variants were responsible for only 4% of familial breast cancer risk, suggesting that increasing samples sizes are allowing the identification of SNPs that confer smaller risks (Michailidou et al., 2017). Up to now, GWAS-identified SNPs collectively explain 18% of the familial breast cancer risk, but it is estimated that this is only 44% of the familial breast cancer risk that can be explained by all imputable SNPs combined (Michailidou et al., 2017). Identification of those SNPs as breast cancer susceptibility alleles will require even larger GWAS sample sizes, but also enrichment of phenotypes associated with breast cancer risk, as SNPs underlying ER-negative breast cancer are currently underrepresented.

In this respect, GWAS has also shown that estrogen receptor (ER)-positive and ER-negative breast cancer share a common etiology as well as a partly distinct etiology. Twenty loci were identified to associate specifically with ER-negative breast cancer, where a further 105 SNPs also associate with overall breast cancer (Milne et al., 2017). Furthermore, there is a common shared etiology for ER-negative breast cancer and breast cancers arising in *BRCA1* mutation carriers as well as overall breast cancer and breast cancer in *BRCA2* mutation carriers (Lilyquist et al., 2018).

Although the risks associated with single GWAS-identified SNPs are low, combining these SNPs in PRSs has shown to be useful for identifying women at high risk for developing breast cancer. In fact, based on a 77-SNP PRS developed by Mavaddat et al. 1% of women with the highest PRS have an estimated 3.4-fold higher risk of developing breast cancer as compared with the women in the middle quintile (Mavaddat et al., 2015). Moreover, PRSs were shown to be particularly useful for risk prediction within carriers of *BRCA1, BRCA2*, and *CHEK2* germline mutations as well as in addition to clinical risk prediction models (Dite et al., 2016; Kuchenbaecker et al., 2017; Muranen et al., 2017).

In summary, GWAS has allowed the research community to be very successful in the identification of risk loci that are associated with genetic predisposition to breast cancer. To date, more than 170 low-risk breast cancer susceptibility alleles have been identified. Unfortunately, for the vast majority of the GWAS-identified risk loci, the causal variant(s), target gene(s) and their functional mechanism(s) have not yet been elucidated (Fachal and Dunning, 2015). Despite the development of tools and strategies for fine-scale mapping and functional analyses, the effort is still huge to characterize each GWAS-identified risk locus and reveal its underlying biology in breast tumorigenesis (Edwards et al., 2013; Fachal and Dunning, 2015; Spain and Barrett, 2015). However, for those 22 breast cancer risk that have been analyzed in more detail, this has provided already significant insight into the, sometimes complex, mechanisms underlying breast cancer susceptibility (Table 1) (Meyer et al., 2008, 2013; Udler et al., 2009, 2010a; Ahmadiyeh et al., 2010; Stacey et al., 2010; Beesley et al., 2011; Cai et al., 2011b; Bojesen et al., 2013; French et al., 2013; Ghoussaini et al., 2014, 2016; Quigley et al., 2014; Darabi et al., 2015, 2016; Glubb et al., 2015; Guo et al., 2015; Lin et al., 2015; Orr et al., 2015; Dunning et al., 2016; Hamdi et al., 2016; Horne et al., 2016; Lawrenson et al., 2016; Shi et al., 2016; Sun et al., 2016; Wyszynski et al., 2016; Zeng et al., 2016; Betts et al., 2017; Helbig et al., 2017; Michailidou et al., 2017).

**Table 1:** Overview of post-GWAS studies that have performed more extensive fine-scale mapping, *in-silico* prediction or functional analysis.

**Locus**	**Putative casual SNPs**	**Target genes**	**DHSS**	**FAIRE**	**TFBS**	**Histone marks**	**3C**	**ChIA-PET**	**EMSA**	**eQTLs**	**Luciferase reporter assay**	**Other**	**References**
1p11.2	2 iCHAVs: rs11249433, rs12134101; rs146784183	*NOTCH2*	rs11249433			H3K27Ac marks at rs11249433				No associations for rs11249433 or rs146784183			Horne et al., 2016
1p34	rs4233486, rs35054111, rs11804913, rs7554973	*CITED4*	rs4233486		JARID1B and FOXM1 bind rs4233486	H3K4Me1, H3K4Me2, H3K4Me3, H3K9Ac, H3K27Ac at rs4233486; H3K4Me1 and H3K27Ac at rs11804913; H3K27Ac at rs7554973	PRE1 and PRE2 interact with the *CITED4* promoter				The rs4233486 risk allele in PRE1 enhances *CITED4* promoter activity		Michailidou et al., 2017
1p36	rs2992756	*KLHDC7A*	rs2992756		ER, PBX1, POLR2A, SPDEF, JARID1B, EP300, FOXA1, GATA3, HIF1α, HIF1β	H3K4Me1, H3K4Me2, H3K4Me3, H3K9Ac, H3K27Ac					The *KLHDC7A* promoter containing the rs2992756 risk allele has reduced activity		Michailidou et al., 2017
2q33	4 iCHAVs: rs1830298, rs10197246; rs36043647; rs59278883; rs7558475	*ALS2CR CASP8 CFLAR*	rs3769823 and rs3769821 in iCHAV1			H3K27Ac marks at rs3769823 and rs3769821 in iCHAV1				minor alleles of rs6754084 and rs6743068 in iCHAV1 decrease *CASP8* expression			Lin et al., 2015
2q35	1 iCHAV: rs4442975, rs6721996	*IGFBP5*	rs4442975		FOXA1 is preferentially recruited to the common allele of rs4442975	H3K4Me1, H3K4Me2 marks near rs4442975	The common allele of rs4442975 interacts with the C allele of chr2:271,557,291 and the *IGFBP5* promoter			The common allele of rs4442975 increases *IGFBP5* expression in ER+ cell lines and normal breast tissue, estrogen induction increases *IGFBP5* expression in cells carrying a common allele of rs4442975	PRE containing rs4442975 does not affect *IGFBP5* expression		Ghoussaini et al., 2014
2q35	1 iCHAV: 14 SNPs including the 1.3 kb enCNV				Enhanced ERα binding on the 1.3 kb enCNV before and after estrogen treatment		1.3 kb enCNV interacts with IGFBP5 promoter					Differential allelic binding of ERα at the 1.3 kb enCNV reduces allele-specific *IGFBP5* expression in response to estrogen	Wyszynski et al., 2016
4q21	89 SNPs	*FAM175A, HELQ, MRPS18C, HSPE*	rs6844460		MAX binds rs6844460	H3K9Ac marks at rs11099601; H3K4Me3, H3K9Ac and H3K27Ac marks at rs6844460		rs11099601 and rs6844460 interact with the *MRPS18C* promoter		The risk allele of rs11099601 associates with decreased *HELQ* and increased *MRPS18C, FAM175A* and *HPSE* expression, but this was inconsistent across data sets			Hamdi et al., 2016
4q24	2 iCHAVs: 24 SNPs; 5 SNPs	*TET2*	rs62331150 and rs73838678 in iCHAV1		rs62331150 in iCHAV1 lies in a SP1, EGR1, NIFC and near a P300 binding site, rs73838678 in iCHAV1 lies in a PR, EGR1, NIFC and near a P300 binding site	H3K4Me and H3K27Ac marks at rs62331150 and H3K27Ac marks are at rs73838678 in iCHAV1		rs62331150 and rs73838678 in iCHAV1 interact with the *TET2* promoter		The risk allele of rs62331150 decreases *TET2* expression			Guo et al., 2015
5p12	rs7716600	*MRPS30*	DHSS cluster at a locus 700 bp from rs7716600	Reduced euchromatic conditions at the *MRPS30* promoter after estrogen stimulation for common homozygotes	2-fold increase in ERα binding at de *MRPS30* promoter and DHSS locus, significant increase in CTCF binding at the *MRPS30* promoter and *CTCF* locus in common homozygotes after estrogen stimulation	The risk allele of rs7716600 associates with decreased methylation of a probe located immediately 5' of *MRPS30*				rs7716600 risk allele upregulates *MRPS30* expression		In ER+ breast tumors *MRPS30* expression correlated strongly with expression of genes in the estrogen signaling pathway. *MRPS30* expression is increased in response to estrogen in MPE600 cells which are homozygous for the risk allele.	Quigley et al., 2014
5p12	3 iCHAVs: rs10941679; 38 SNPs; rs200229088	*FGF10, MRPS30, HCN1*			FOXA1 and OCT1 bind rs10941679, but not allele specific	None	*FGF10* and *MRPS30* promoter		allele-specific binding of rs10941679 by FOXA1, FOXA2, CEBPB and OCT1	rs10941679 risk allele upregulates *FGF10* and *MRPS30* expression	rs10941679 risk allele had no additional effect on the PRE enhancer activity for *FGF10, MRPS30* and *BRCAT54* promoters		Ghoussaini et al., 2016
5p15.33	rs2736108, rs2736109	*TERT*								None	rs2736108 and rs2736109 risk alleles combined reduce *TERT* promoter activity		Beesley et al., 2011
5p15.33	3 iCHAVs: 7 SNPs, 6 SNPs, 3 SNPs	*TERT*	None	600–800 bp of open chromatin covering rs10069690 and 2242652	None	None					rs2736107, rs2736108 and rs2736109 minor alleles from iCHAV1 decreased transcription; including the rs7705526 minor allele from iCHAV2 increases *TERT* promoter activity; the PRE and rs2242652, but not rs10069690 from iCHAV3 decrease *TERT* promoter activity	The rs10069690 minor allele associates with an alternatively spliced *TERT* isoform leading to a premature stop codon	Bojesen et al., 2013
5p15.33	rs3215401, rs2853669	*TERT*		rs2736108 and rs2736109 common alleles, but not rs3215401 and rs2853669 alleles associated with open chromatin	SP2, ZTTB7A for rs3215401 and ETS, MYC, MIXL1, RBPJ, SIN3A, ZNF143, EP300 for rs2853669; ChIP for GABPA and MYC, but not ETS2, ELF1 or E2F1 led to preferential isolation of the rs2853669 risk allele		None				rs3215401 and rs2853669 risk alleles reduce *TERT* promoter activity, but not rs2736107, rs2736108, rs2736109 and rs145544133 risk alleles	Silencing of MYC, but not ETS2 downregulated *TERT* promoter activity irrespective of rs2853669 genotype	Helbig et al., 2017
5q11.2	3 iCHAVs: 15 SNPs; 90 SNPs, 66 SNPs; 5 SNPs	*MAP3K1*			FOXA1 binds PRE-B1, ERα binds PRE-C, GATA3 preferentially bind the risk allele of iCHAV3 rs17432750 in PRE-B3	H3K4Me1, H3K4Me2, H3K27Ac	*MAP3K1* promoter	All 4 PREs interact with the *MAP3K1* promoter		No association	In iCHAV1 PRE-A downregulates *MAP3K1* and PRE-B1 and PRE-C upregulate *MAP3K1*, rs74345699 and rs62355900 risk alleles in PRE-C further upregulate *MAP3K1* in the presence of estrogen; in iCHAV2a PRE-D upregulates *MAP3K1*, which is further enhanced by the rs16886397 risk allele; in iCHAV2b PRE-2B upregulates *MAP3K1*, which is further enhanced by the rs62355881 risk allele; in iCHAV3 PRE-B3 downregulates *MAP3K1*, which is further reduced by the rs17432750 risk allele	siRNA against *GATA3* reduced the enhancer activity of PRE-B3 containing the risk allele of rs17432750	Glubb et al., 2015
6q25.1	rs9397435, rs77275268	*ESR1, PGR*			None for rs9397435, CTCF for rs77275268	H3K4Me1, H3K4Me2, H3K9Ac for rs9397435				rs9397435 risk homozygotes show increased *ESR1* and *PGR* expression		rs77275268 is located in a partially methylated CpG sequence	Stacey et al., 2010
6q25.1	rs6913578, rs7763637				None				The risk allele of rs6913578 significantly altered DNA-protein complex intensity, no detectable interaction of rs7763637 with nuclear proteins		Transcription activation was significantly increased for common alleles of rs6913578 and rs7763637		Cai et al., 2011b
6q25.1	rs7763637	*AKAP12, ESR1, RMND1, ZBTB2*			ZNF217, FOS, KAP1, JUND, FOSL2, JUN, MYC	H3K4Me3, H3K4Me1, H3K27Ac				rs7763637 risk allele upregulates *AKAP12* expression in adjacent normal breast tissue and breast tumors, but downregulates *ESR1, RMND1* and *ZBTB2* in breast tumors			Sun et al., 2016
6q25.1	5 iCHAVs: 10 SNPs; 3 SNPs; 4 SNPs; 3 SNPs; 6 SNPs	*ESR1, RMND1, CCDC170*	19 of the 26 candidate causal SNPs		GATA3 binds the risk allele of iCHAV3 SNP rs851982; CTCF binds the risk allele of iCHAV3 SNP rs851983 and the common allele of iCHAV4 SNP rs1361024; MYC binds the common allele of iCHAV5 SNP rs910416	19 of the 26 candidate causal SNPs were associated with enhancer enriched histone marks; H3K27Ac marks were enriched at rs2046210, rs12173570 and rs851984	iCHAV1-2 elements interact with *ESR1, RMND1-ARMT1* and *CCDC170* promoters; iCHAV3-5 elements interact with *ESR1* and *RMND1-ARMT1* promoters; the common allele of iCHAV4 SNP rs1361024 increases looping to *ESR1* and *RMND1* promoters		11 of the 19 causal candidate SNPs associated with PREs, altered the binding activity of transcription factors of which 7 fell within promoter-specific interactions as identified by 3C	Risk alleles of iCHAV1 reduced ER expression; risk alleles of iCHAV1, 3 and 5 increased *ESR1* expression in ER+ tumors compared with normal, tumor-adjacent tissue; Imbalance in allele-specific expression in *ESR1* for SNPs in iCHAV1-3, in *CCDC170* for iCHAV2 SNP rs9397437 and in *RMND1* for iCHAV3 SNP rs851983; risk alleles of iCHAV3 associate with *CCDC170* expression	iCHAV1 SNP rs6557160, iCHAV2 SNP rs17081533 and iCHAV5 SNP rs910416 reduce *ESR1* and *RMND1* promoter activity, although inclusion of the iCHAV1 haplotype reduced *ESR1, RMND1* and *CCDC170* promoter activity; iCHAV3 SNP rs851982 increased *ESR1* promoter activity		Dunning et al., 2016
7q22	rs13229095, rs6979850, rs6961094, rs7796917, rs71559437, rs11972884	*CUX1, RASA4, PRKRIP1*	rs4233486		CEBPB, ER, FOXA1, FOXM1, E2F1, MAX, P300, PBX1, SIN3A, MYC, SPDEF, FOSL2, GATA3, NR2F2, RARA, TCF7L2, POLR2A, REST, RIP140	H3K4Me1, H3K4Me2, H3K4Me3, H3K9Ac, H3K27Ac	PRE1 interacts with the *CUX1* and *RASA4* promoter; PRE2 interacts with the *RASA4* and *PRKRIP1* promoter; the risk haplotype associated with chromatin looping						Michailidou et al., 2017
8q24						H3K4Me2	*MYC*						Ahmadiyeh et al., 2010
8q24	5 iCHAVs: rs35961416; rs13281615; rs7815245; rs2033101; rs1121948		rs7815245; rs1121948		The rs7815245 risk allele alters the TCF12 binding motif and is located in an ESR1 and close to a FOXA1 binding site; rs1121948 is located in a GATA3 and MAX binding site	H3K4Me1, H3K27Ac marks at rs1121948				The rs7815245 risk allele downregulates *POU5F1B*; the rs1121948 risk allele downregulates *PVT1* and *MYC*			Shi et al., 2016
9q31.2	3 iCHAVs: 28 SNPs; rs10816625; rs13294895	*KLF4*	iCHAV1: rs662694, rs471467, rs5899787		CTCF binds iCHAV1 rs662694 and rs471467, ERα, FOXA1, FOXM1 and GATA3 bind iCHAV1 rs5899787; ERα, FOXA1, FOXM1, GATA3, HDAC2, Max, NR2F2, P300 and Sin3A bind iCHAV2 rs10816625 and iCHAV3 rs13294895	iCHAV1 rs5899787 site is enriched for H3K27me3 marks; iCHAV2 rs10816625 and iCHAV3 rs13294895 localize to a PRE marked by H3K27Ac and H3K4Me1	iCHAV2 rs10816625 and iCHAV3 rs13294895 interact with the *KLF4* promoter				iCHAV2 rs10816625 and iCHAV3 rs13294895 decrease *KLF4* expression		Orr et al., 2015
10q21	4 iCHAVS: 12 SNPs; 17 SNPs; 18 SNPs; rs9971363, rs7090365	*ZNF365 NRBF2*	PRE1 and PRE2 in iCHAV2			H3K4Me1 and H3K4Me2 marks are enriched at PRE1 and PRE2 in iCHAV2	iCHAV2 interacts with *ZNF365* and *NRBF2* promoters			No association	iCHAV2 protective haplotype downregulates *NRBF2* and *ZNF365* expression		Darabi et al., 2015
10q26	rs7895676, rs2981578	*FGFR2*			C/EBPβ, RUNX2				C/EBPβ binds rs7895676 minor allele, RUNX2 binds rs2981578 minor allele	Minor alleles of rs7895676 and rs2981578 upregulate *FGFR2*	No significant transcription activation for minor allele of rs7895676, but synergizes with rs2981578; 2-5 fold higher transcription activation for minor allele of rs2981578		Meyer et al., 2008
10q26	3 iCHAVs: rs35054928, rs34032268, rs2981579, rs2912779, rs2912780; rs45631563; rs2981578, rs45631539	*FGFR2*	rs35054928, rs2981579, rs2912779; rs45631563; rs2981578	Increased chromatin accessibility of the risk allele	E2F1 preferentially binds rs35054928 minor allele, no enrichment for SP1; Ser5P-Pol II, FOXA1 and ERα preferentially bind rs2981578 minor allele, low enrichment for RUNX2		*FGFR2* promoter	*FGFR2* promoter	E2F1 and SP1 bind rs35054928, ERα binds rs2981579, an unidentified protein binds to rs2912779; an unidentified nuclear protein binds rs45631563; OCT1, RUNX2 and FOXA1 bind rs2981578	No association between rs35054928 or rs2981578 genotypes and *FGFR2, ATE1, NSMCE4A* or *TACC2* expression		siRNA against *FOXA1* downregulates FGFR2, siRNA against *E2F1* had little effect on FGFR2 but upregulated FOXA1	Meyer et al., 2013
10q26	rs7895676, rs10736303, rs2912781, rs2912778, rs2981578		rs2912778, rs2981578										Udler et al., 2009
11p15	19 SNPs	*PIDD1*	chr11:801630_-_ATG, rs7104785, rs7484123, rs7484068, rs11246313, rs11246314		ER, NR2F2, RIP140, SPDEF, CTCF, E2F4, POLR2A, EGR1, GABPA, E2F1, JARID1B, PML, FOXM1, EGLN2, HIF1α, HIF1β, NRF1	H3K4Me1, H3K4Me2, H3K4Me3, H3K9Ac, H3K27Ac					The *PIDD1* promoter containing the risk haplotype has increased activity		Michailidou et al., 2017
11q13	3 iCHAVs: rs661204, rs78540526, rs554219, rs657686; rs75915166; rs494406, rs585568, rs593679, rs679162	*CCND1*			The 4 iCHAV1 SNPs fall in PRE1 which binds ERα and FOXA1, allele-specific binding of ELK4 to rs554219; iCHAV2 SNP rs75915166 falls in PRE2	PRE1 is flanked by H3K4Me1 and H3K4Me2 marks	PRE1 interacts with the *CCND1* promoter and an enhancer of *CCDN1* located in the *CCDN1* terminator region; PRE2 interacts with the *CCDN1* promoter; PRE1 interacts with PRE2	PRE1 interacts with the *CCND1* promoter and an enhancer of *CCDN1* located in the *CCDN1* terminator region	The common alleles of rs661204 and rs78540526 preferentially bind USF1 and USF2, the common allele of rs554219 is bound by ELK4 and GABPA; the minor allele of rs75915166 interacts specifically with GATA3	homozygotes for the rs554219 risk allele have reduced cyclin D1 expression	Risk alleles of rs78540526 and rs554219 abolish PRE1 enhancer activity and decrease *CCND1* promoter activity, PRE1 is estrogen inducible irrespective of the SNPs genotypes; minor allele of rs75915166 increases strength of PRE2 silencer	siRNA against ELK4 reduce enhancer activity of wild type PRE1, but not PRE1 containing the risk allele of rs554219; siRNA against *GATA3* increases transcription in the presence of the rs75915166 risk allele, but not the common allele	French et al., 2013
11q13		*CCND1, CUPID1, CUPID2*					the risk alleles of rs661204 and rs78540526 abolish interaction of PRE1 with the predicted promoter of *CUPID1* and *CUPID2*				risk alleles of rs661204 and rs78540526 reduced PRE1 enhancer activity on the *CUPID1* promoter	silencing PRE1 by dCas9-KRAB reduced *CUPID1, CUPID2* and *CCND1* expression, estrogen induction of the *CUPID1* and *CUPID2* promoter depended on PRE1 but not the risk SNPs, *CUPID1* and *CUPID2* regulated genes affect DNA repair and recombination, tumors with low *CCND1, CUPID1* or *CUPID2* expression had similar mutational signatures as BRCA1 and BRCA2 deficient tumors, silencing of *CUPID1* and *CUPID2* impaired end resection and NHEJ could compensate for the lack of HRR	Betts et al., 2017
12p11	4 iCHAVs: 4 SNPs; 74 SNPs; 376 SNPs; 2 SNPs	*CCDC91, PTHLH*			iCHAV1 rs812020 disrupts a E2F3 binding site; iCHAV2 rs788463 is in a C/EBP binding site and rs10843066 disrupts a HNF1B binding site; iCHAV3 rs10843110 disrupts a PPARG binding site and rs11049453 disrupts a PAX binding site	H3K4Me3 and H3K27Ac at iCHAV1-4		multiple iCHAV1 and iCHAV2 SNPs interact with the *PTHLH* promoter		The iCHAV3 rs11049453 risk allele increases *PTHLH* and decreases *CCDC91* expression			Zeng et al., 2016
16q12	14 SNPs	*TOX3, LOC643714*	rs12930156, rs3095604, rs45538731, rs28463809, rs4784226		The risk allele of rs4784227 creates a C/EBPα binding site					No association between rs3803662 genotypes and *TOX3* expression; rs3803662 genotypes associated with *RBL2* expression in lymphocytes, but not breast tumors			Udler et al., 2010b
17q22	28 SNPs	*STXBP4*	rs244353, rs2787481, rs244371			None		No interactions		rs2787481 genotypes associate with *COX11* expression; rs2787481, rs244317 and rs11658717 genotypes downregulate *STXBP4* and upregulate a short *STXBP4* isoform, rs244353 genotypes downregulate *STXBP4* expression		rs244353 is located in an enhancer predicted to target the *STXBP4* gene and an enhancer predited to target the *HLF* gene	Darabi et al., 2016
19p13.1	1 iCHAV: 13 SNPs	*ABHD8, ANKLE1*			rs55924783 and rs56069439 coincided with CTCF binding sites	rs56069439 and rs4808616 coincided with H3K4Me1 marks	rs4808075, rs10419397, rs56069439 and rs4808076 interact with the *ABHD8* promoter			The risk allele of 13 SNPs associate with increased *ABHD8* expression; the risk allele of rs56069439 associates with greater allele-specific expression of *ABHD8*	PRE-A, B and C upregulate *ABHD8*, which is further enhanced by the risk alleles of rs56069439, rs113299211, rs67397200, rs61494113, rs4808616 and rs55924783; PRE-A silences *ANKLE1*; PRE-C upregulates *ANKLE1*, which is reduced by the risk alleles of rs4808616 and rs55924783	CRISPR/Cas9 deletion of a 57 bp region containing rs56069439 reduced *ANKLE1*, but not *ABHD8* or *BABAM1* expression; overexpression of *ABHD8* reduced cell migration and invasion and caused expression changes in cancer-related pathways, overexpression of *ANKLE1* caused expression changes in cancer-associated and cell growth/proliferation pathways	Lawrenson et al., 2016

*DHSS, DNase I hypersensitivity sites; FAIRE, Formaldehyde-assisted isolation of regulatory elements; TFBS, Transcription factor binding sites; 3C, Chromatin conformation capture; ChIA-PET, Chromatin interaction analysis by paired-end tag sequencing; EMSA, Electrophoretic mobility shift assay; eQTLs, Expression quantitive trait loci; Ref, reference; iCHAV, Independent set of correlated highly associated variants; enCNV, Enhancer copy number variation; PRE, Putative regulatory element; NHEJ, Non-homologous end joining; HRR, homologous recombination repair*.

## Fine-scale mapping of GWAS-identified loci

GWAS-identified SNPs usually do not represent the causal risk variants. These are merely tags to a locus associated with risk for developing the disease. However, because each causal variant is located in a region containing an independent set of correlated highly associated variants (iCHAV) (Edwards et al., 2013), fine-scale mapping of GWAS-identified loci in large sample sizes is required in order to identify the causal variant from a background of non-functional highly correlated neighboring SNPs.

In order to fulfill successful fine-scale mapping, a complete list of all SNPs, including the causal variants, should be available for the risk locus of interest. Direct sequencing of the risk locus would be a good approach for achieving this, however, it is an expensive method. Particularly since successful fine-scale mapping requires sufficient statistical power and thus sample sizes up to 4-fold to that of the original GWAS (Udler et al., 2010b). In this respect, the 1000 genome project containing whole genome sequencing data of 2,504 individuals from 26 populations is a valuable resource (Auton et al., 2015; Zheng-Bradley and Flicek, 2017). A second prerequisite for successful fine-scale mapping is large sample sizes, which are usually only achieved within large consortia such as BCAC. Therefore, both the iCOGS array as well as the OncoArray, in addition to a GWAS backbone, additionally contained numerous SNPs for fine-scale mapping of previously GWAS-identified risk loci (Michailidou et al., 2013, 2017).

Once a dense set of SNPs for a given GWAS-identified risk locus has been genotyped statistical analyses are applied to reduce the number of candidate causal SNPs. Interestingly, it seems to be a common theme among GWAS-identified loci that the underlying risk is conferred by more than one iCHAV. For breast cancer risk loci at 1p11.2, 2q33, 4q24, 5p12, 5p15.33, 5q11.2, 6q25.1, 8q24, 9q31.2, 10q21, 10q26, 11q13, and 12p11 multiple iCHAVs have been identified ranging from two to a maximum of five iCHAVs at 6q25.1 and 8q24 (Table 1) (Bojesen et al., 2013; French et al., 2013; Meyer et al., 2013; Darabi et al., 2015; Glubb et al., 2015; Guo et al., 2015; Lin et al., 2015; Orr et al., 2015; Dunning et al., 2016; Ghoussaini et al., 2016; Horne et al., 2016; Shi et al., 2016; Zeng et al., 2016). For this reason, the first step in the fine-scale mapping process is establishing how many iCHAVs are present at a particular GWAS-identified risk locus using forward conditional regression analysis (Edwards et al., 2013). Then for each iCHAV, the SNP displaying the strongest association with breast cancer risk is identified. Based on this SNP, other SNPs within the same iCHAV are excluded from being candidate causal variants when the likelihood ratio for that SNP is smaller than 1:100 in comparison with the SNP showing the strongest association (Udler et al., 2010b). The reduction in candidate causal variants that is achieved during this process not only depends on sample size, but also the LD structure of the GWAS-identified locus.

Importantly, the majority of GWAS-identified risk loci were discovered in populations of European ancestry. Because the LD structure of the European ancestry population shows larger LD blocks containing more highly correlated SNPs than Asian or African ancestry populations, this offers an advantage in GWAS studies since less tagging SNPs are needed to achieve genome-wide coverage. However, for fine-scale mapping this is disadvantageous since the large number of highly correlated variants within an iCHAV may not allow sufficient reduction of candidate causal variants (Edwards et al., 2013). Therefore, fine-scale mapping in additional populations besides the European ancestry population (i.e., Asian and African ancestry populations) can be an effective strategy to reduce the number of candidate causal variants from iCHAVs located at GWAS-identified regions and add validity to the remaining candidate causal SNPs (Stacey et al., 2010; Edwards et al., 2013). Requirements for success are sufficient sample sizes for all populations, different correlation patterns between the studied populations and the risk association must be detectable in the additional populations, which usually depends on the risk allele frequency in these populations (Edwards et al., 2013). Unfortunately, the LD structure at the GWAS-identified risk loci is not always favorable and multiple highly correlated candidate causal variants remain. In this respect, analysis of the haplotypes that are present in a particular population and evaluation of their association with breast cancer risk may provide another strategy for exclusion of non-causal SNPs within an iCHAV (Chatterjee et al., 2009).

The purpose of fine-scale mapping is to identify the number of iCHAVs underlying GWAS-identified risk loci and reducing the number of candidate causal variants in these iCHAVs to a minimum. In practice, this reduction does not directly lead to identification of the single causal variant responsible for this risk due to several of the reasons described above. Either way, whether only one, a few or many candidate causal SNPs remain, in the next phase the candidate causal variants need to be validated or further reduced by elucidating the functional mechanism through which these variants operate. First, overlap between the candidate causal variants and regulatory sequences such as transcription factor (TF) binding sites, histone marks or regions of open chromatin is evaluated *in silico*. In addition, expression quantitative trait loci (eQTL) studies are performed in order to identify the genes that are deregulated by the candidate causal variants. The hypotheses for the functional mechanisms by which the candidate causal SNPs confer breast cancer risk are then further tested by molecular experiments in *in-vitro* model systems.

## *In-silico* prediction of functional mechanisms

The vast majority of GWAS-identified SNPs are not protein-coding and are located in intronic or intragenic regions, or even in gene deserts (www.genome.gov/gwastudies). Their underlying causal variants usually have a regulatory role by modulating the expression of target genes or non-coding RNAs (ncRNAs). Therefore, causal variants usually coincide with regulatory regions associated with open chromatin, TF binding sites, sites of histone modification or chromatin interactions (Table 1) (Meyer et al., 2008, 2013; Stacey et al., 2010; Udler et al., 2010a; Beesley et al., 2011; Cai et al., 2011a; Bojesen et al., 2013; French et al., 2013; Ghoussaini et al., 2014, 2016; Quigley et al., 2014; Darabi et al., 2015, 2016; Glubb et al., 2015; Guo et al., 2015; Lin et al., 2015; Orr et al., 2015; Dunning et al., 2016; Hamdi et al., 2016; Lawrenson et al., 2016; Shi et al., 2016; Sun et al., 2016; Wyszynski et al., 2016; Zeng et al., 2016; Betts et al., 2017; Helbig et al., 2017; Michailidou et al., 2017). Mining public data for these regulatory features can be an effective way to narrow down the list of candidate causal variants after fine-scale mapping. Furthermore, to determine which candidate causal SNPs affect gene expression, eQTLs can be evaluated. Besides narrowing down the list of candidate causal variants, these *in silico* predictions, additionally, provide clues about the functional mechanisms involved, which will guide the design of molecular experiments.

### Regulatory features

A wealth of data is publically available regarding regulatory features throughout the genome. Via ENCODE (https://www.encodeproject.org/), data on locations of open chromatin, TF binding sites, DNA methylation, RNA expression and histone modifications can be retrieved (Djebali et al., 2012; ENCODE Project Consortium, 2012; Neph et al., 2012; Sanyal et al., 2012; Thurman et al., 2012). The NIH Roadmap Epigenomics project (http://www.roadmapepigenomics.org/) contains data on locations of open chromatin, DNA methylation and histone modifications (Kundaje et al., 2015; Zhou et al., 2015). In addition, Nuclear Receptor Cistrome (http://cistrome.org/NR_Cistrome/index.html) also has information on TF binding locations. Using FunctiSNP (http://www.bioconductor.org/packages/release/bioc/html/FunciSNP.html), RegulomeDB (http://www.regulomedb.org/) and HaploReg (http://archive.broadinstitute.org/mammals/haploreg/haploreg.php) these sources of information can be mined allowing the prediction of putative regulatory regions (PREs) within an iCHAV (Boyle et al., 2012; Coetzee et al., 2012; Ward and Kellis, 2012). The long range chromatin interactions that these PREs may establish can subsequently be assessed via GWAS3D (http://jjwanglab.org/gwas3d) and the 3D Genome Browser (http://promoter.bx.psu.edu/hi-c/) providing clues about the target genes or ncRNAs that could be deregulated (Li et al., 2013a; Yardimci and Noble, 2017).

Interestingly, several regulatory features appear to be enriched among GWAS-identified breast cancer risk loci, such as TF binding sites for ERα, FOXA1, GATA3, E2F1, and TCF7L2, but also H3K4Me1 histone marks as well as regions of open chromatin marked by DNAse I hypersensitivity sites (DHSSs) (Cowper-Sal lari et al., 2012; Michailidou et al., 2017). It is important to keep in mind, however, that despite of the wealth of data available, these data sources harbor information for only a fraction of the TFs present in the human proteome. This means that other regulatory features, which we are currently unable to evaluate, may also play an important role in mediating the susceptibility to breast cancer. Moreover, TFs, as well as histone marks and chromatin interactions, are highly tissue specific and it will therefore be crucial to evaluate these regulatory features in the proper tissue type or cell line to prevent either false positive or false negative associations. In order to obtain a more comprehensive understanding of the mechanisms underlying breast cancer predisposition, we thus need cistrome data on more TFs from more tissue types.

Still, mining of the currently available data has facilitated the identification of causal variants and/or functional mechanisms for several of the identified GWAS-identified loci (Meyer et al., 2008, 2013; Udler et al., 2010a; French et al., 2013; Ghoussaini et al., 2014, 2016; Quigley et al., 2014; Darabi et al., 2015; Glubb et al., 2015; Guo et al., 2015; Orr et al., 2015; Dunning et al., 2016; Hamdi et al., 2016; Lawrenson et al., 2016; Shi et al., 2016; Zeng et al., 2016; Helbig et al., 2017; Michailidou et al., 2017). Combining information on regulatory features from candidate causal variants with eQTLs will further narrow down the list of candidate variants, identify target genes and provide a starting point for subsequent *in-vitro* molecular experiments.

### eQTLs

eQTLs are variants that control gene expression levels and are therefore found in regulatory regions in the genome. Evidence for a candidate causal variant to be associated with gene expression can be obtained from eQTL studies. In an eQTL study, the presence of a correlation between expression levels of potential target genes and the genotypes of the candidate causal variants is evaluated in an unbiased manner. Two types of eQTL studies are generally distinguished based on the distance of the gene from the candidate SNP. In cis-eQTL studies, the target genes being evaluated are in close proximity to the candidate causal variant, usually within 1 to 2 megabases. For trans-eQTL studies, all genes outside this region, thus also on other chromosomes, are subjected to evaluation (Cheung and Spielman, 2009). Far more genes are thus tested for correlation with candidate causal variants in trans-eQTL analyses than cis-eQTL analyses and, consequently, trans-eQTL studies require far more statistical power than cis-eQTL studies. It is therefore that in most of the post-GWAS analyses only cis-eQTL analysis is performed. Moreover, besides gene expression, eQTLs can also influence the expression of ncRNAs, mRNA stability, differences in allelic expression and differential isoform expression (Ge et al., 2009; Lalonde et al., 2011; Pai et al., 2012; Kumar et al., 2013).

SNPs that are located in regulatory regions of genome show a higher tissue specificity and it is therefore no surprise that eQTLs in GWAS-identified regions also display high tissue specificity (Dimas et al., 2009; Fu et al., 2012). Consequently, choice of tissue type in an eQTL study is critical to prevent false positive or false negative associations. The most obvious choice is the target tissue under investigation. For breast cancer, this can be either normal breast tissue or breast tumor tissue. In this respect, the cancer genome atlas (TCGA; https://cancergenome.nih.gov/), Molecular Taxonomy of Breast Cancer International Consortium (METABRIC; http://www.ebi.ac.uk/ega/) and Genotype Tissue Expression (GTEx; https://gtexportal.org/home/) are valuable resources (Cancer Genome Atlas Network, 2012; Curtis et al., 2012; Battle et al., 2017). However, eQTL studies in breast cancer tissue are confounded by the presence of copy number variation, somatic mutations and differential methylation that influence gene expression levels. Therefore, eQTLs are ideally evaluated in normal breast tissue. Unfortunately, availability of both genotyping and gene expression data for normal breast tissue is limited as compared with breast tumor tissue, resulting in lower statistical power in eQTL analyses. Alternatively, for breast tumor analyses, gene expression data could also be adjusted for somatic CNVs and methylation variation (Li et al., 2013b). In addition, it should also be considered that the tumor micro-environment plays an important role in the development of breast cancer and that expression levels deregulated in stroma or immune cells might also be relevant.

It is important to treat the identification of eQTLs with some caution. False positives and false negatives could be a result from choosing the incorrect tissue type. In six post-GWAS studies to date an eQTL association was observed and an attempt was made to validate these results with luciferase reporter assays (Meyer et al., 2008; French et al., 2013; Ghoussaini et al., 2014, 2016; Dunning et al., 2016; Lawrenson et al., 2016). For GWAS-identified risk loci at 2q35 and 5p12, luciferase reporter assays did not confirm the eQTL association, whilst this was the case for eQTL associations at 6q25.1, 10q26, 11q13, and 19q13.1 (Table 1). In addition, when evaluating cis-eQTLs, false negative results could also imply that more distant eQTLs are involved. Moreover, since causal variants from different iCHAVs within a GWAS-identified region can influence the same target gene (Bojesen et al., 2013; French et al., 2013; Glubb et al., 2015; Dunning et al., 2016; Lawrenson et al., 2016), eQTLs may remain undetected. For example, in the post-GWAS study by Glubb et al. at the 5q11.2 locus, PRE-A downregulated *MAP3K1*, whereas PRE-B1 and PRE-C upregulated *MAP3K1* expression although no eQTL associations were identified (Glubb et al., 2015). Similarly, Lawrenson et al. studied the GWAS-identified breast cancer risk locus at 19p13.1 and noticed PRE-A downregulating *ANKLE1* and PRE-C upregulating *ANKLE1* expression, while no eQTL association was detected. Interestingly, at this same locus three PREs regulating *ABHD8* all upregulated its expression and consistent with this 13 eQTL associations were detected of which one was allele-specific (Lawrenson et al., 2016). Thus, absence of an association does not necessarily imply trans-eQTL associations. For the above reasons, additional *in vitro* molecular experiments are necessary to confirm the results from eQTL studies, but also from the *in silico* predictions of regulatory features and chromatin interactions.

A recently developed tool that is also of interest to predict target genes from GWAS-identified breast cancer risk loci is INQUISIT (integrated expression quantitative trait and *in silico* prediction of GWAS targets) which combines both regulatory features and eQTL data from publically available resources (Michailidou et al., 2017). Interestingly, INQUISIT predicted target genes for 128 out of 142 GWAS-identified breast cancer risk loci and among the 689 target genes a strong enrichment was observed for breast cancer drivers. Furthermore, pathway analysis of these genes revealed involvement of fibroblast growth factor, platelet-derived growth factor and Wnt signaling pathways to be involved in genetic predisposition to breast cancer as well as the ERK1/2 cascade, immune response and cell cycle pathways (Michailidou et al., 2017). However, the expression of breast cancer driver genes is not necessarily deregulated in the same direction by the germline variants as by somatic mutations. For example, *MAP3K1* is upregulated and *CCND1* and *TERT* are downregulated in the germline. This is in contrast with breast tumors, where *MAP3K1* is downregulated and *CCND1* and *TERT* are upregulated by somatic mutations (Bojesen et al., 2013; French et al., 2013; Glubb et al., 2015).

## *In-vitro* functional experiments

After *in silico* prediction of regulatory features and the identification of putative target genes, results should be validated by molecular experiments and the working hypotheses of the mechanistic model should be tested. The model system for these molecular experiments are commonly normal breast or breast cancer cell lines. This is because cell lines can easily be maintained and manipulated. Furthermore, they represent an unlimited source of cells and are generally well characterized (Hollestelle et al., 2010a). The advantage of breast cancer cell lines is that many are available with different characteristics, however, as with eQTL analysis, CNVs, somatic mutations and methylation may be confounding the results of the experiments. Furthermore, for studying the effects of germline variants in breast cancer predisposition and considering that these are likely early events in tumorigenesis, normal breast cell lines seem the obvious choice. Currently two normal breast cell lines have been used in post-GWAS analysis, MCF10A and Bre-80 (Darabi et al., 2015; Glubb et al., 2015; Dunning et al., 2016; Ghoussaini et al., 2016; Lawrenson et al., 2016; Betts et al., 2017; Helbig et al., 2017). Both normal breast cell lines are, however, ER-negative which may not be the best model system for studying candidate causal variants in iCHAVs that are only associated with ER-positive breast cancer. Because of tissue specificity the compromise would therefore be to at least use one normal breast cancer cell line and two breast cancer cell lines, one ER-positive and one ER-negative.

### Chip assays and EMSA

In order to validate the *in silico* predictions of regulatory functions, such as TF binding to a candidate causal SNP or PRE, but also its allele-specific binding, two different techniques can be used. The first is a chromatin immunoprecipitation (ChIP) assay in which antibodies are used to enrich DNA fragments bound by one specific protein. The ChIP is subsequently followed by either sequencing, a qPCR or an allele-specific PCR to identify where a particular TF binds and whether this is allele-specific (Collas, 2010). The second is an electrophoretic mobility shift assay (EMSA) in which a protein or protein extract is mixed with a particular DNA fragment and incubated to allow binding. This mixture is subsequently separated by gel electrophoresis and compared to the length of the probe without protein. When protein binds to the DNA fragment, this results in an upward shift of the gel band. Although this does not provide any clue about the proteins involved in binding the DNA fragment, this assay can be adapted to a super shift assay by adding antibodies against TFs of interest to the protein-DNA mixtures (Hellman and Fried, 2007).

The advantage of ChIP assays is that they produce reliable results for assessing allele-specific binding of TF, in contrast to EMSAs. However, ChIP assays are relatively expensive and the resolution for determining the binding site is low (Edwards et al., 2013). In the post-GWAS analysis at 6q25.1 by Dunning et al. both EMSAs and ChIP assays were performed (Table 1). In this study, a total of five iCHAVs were identified containing 26 candidate causal variants using fine-scale mapping. *In silico* analyses showed that 19 of these candidate causal variants were located in DHSSs. Then, using EMSAs, 11 of these 19 variants were shown to alter the binding affinity of TFs *in vitro*. In the end, the TF identity for four of these candidate causal variants could be established and they appeared to be GATA3, CTCF, and MYC. With ChIP, the authors then confirmed GATA3 binding to iCHAV3 SNP rs851982. Moreover, CTCF binding was enriched at the common allele of iCHAV4 rs1361024, suggesting allele-specific binding of CTCF at this locus (Dunning et al., 2016).

### 3C and ChIA-PET

To validate *in silico* predictions of chromatin interactions or to confirm results from eQTL studies, molecular experiments such as chromatin confirmation capture (3C) can be performed. Using 3C, loci that are physically associated through chromatin loops are ligated together and these ligation products can subsequently be quantified using qPCR (Dekker et al., 2002). In addition, the ligation products can also be sequenced. This way, allele-specific chromatin interactions can be identified. For validating specific chromatin interactions, 3C is a very suitable technique as shown by its wide use in post-GWAS studies (Table 1). However, there are of course also some disadvantages to 3C. One of these is that the background is high at short distances between the two interacting loci. Consequently the two loci under evaluation should be further than 10 kb apart (Monteiro and Freedman, 2013). For instance, in the post-GWAS study at the 19p13 region by Lawrenson et al., only five from the 13 candidate causal variants could be evaluated due to the close proximity of these variants to their target gene, *ANKLE1* (Lawrenson et al., 2016). Usually, this however does not present a problem, since three quarters of distal PREs influences a gene that is not the nearest one (Sanyal et al., 2012).

Another technique that is important to mention in this respect is chromatin-interaction analysis by paired-end tag sequencing (ChIA-PET). This is an adaptation of the original 3C technique allowing the detection of chromatin interactions bound by a specific protein, using an antibody (Fullwood et al., 2009). Usually, ChIA-PET experiments are not specifically performed for each separate post-GWAS study. Because the data is genome-wide, it is usually mined from databases containing interactomes for the most common TFs and histone marks such as ER, CTCF, RNA polymerase II and H3K4Me2. As with the publically available data from cistromes, as discussed earlier, having ChIA-PET data from more cell types and more TFs will improve upon the value of these data for the research community.

### Luciferase reporter assays and CRISPR/Cas9 genome editing

By now, having compiled all *in silico* data and data from molecular experiments, a working hypothesis should be established of how the candidate causal variants confer breast cancer risk. This model includes which candidate causal variant via what TF can modulate gene expression of that particular gene via chromatin interaction. The last step is then usually to conduct luciferase reporter assays in order to confirm this hypothesis and assess what impact the candidate causal variants have on the promoter of that target gene, either enhancing or repressive.

In luciferase reporter assays, PREs are cloned into a reporter construct that expresses the *luciferase* cDNA when the promoter of interest is activated (Gould and Subramani, 1988; Williams et al., 1989; Fan and Wood, 2007). It is common to first establish a baseline for luciferase expression from the wild-type PREs. After that, PREs containing the risk allele or risk haplotype for one or more candidate causal variants are assessed, usually per PRE or per iCHAV. Depending on the levels of luciferase expression after introduction of the risk allele(s), an enhancing or repressive effect can be determined. Moreover, by varying the size of the PREs in subsequent experiments the boundaries of the PRE can be better defined. As discussed before, again the choice of cell type is also relevant here as well as the choice of promoter to use.

For most of the post-GWAS breast cancer risk loci, luciferase reporter assays were performed to confirm the working hypothesis for the functional model (Table 1) (Meyer et al., 2008; Beesley et al., 2011; Cai et al., 2011b; Bojesen et al., 2013; French et al., 2013; Ghoussaini et al., 2014, 2016; Darabi et al., 2015; Orr et al., 2015; Dunning et al., 2016; Lawrenson et al., 2016; Betts et al., 2017; Helbig et al., 2017; Michailidou et al., 2017). However, at the 2q35 locus in the study by Ghoussaini *et al*., the PRE did not influence *IGFBP5* expression despite positive 3C and eQTL results (Ghoussaini et al., 2014). Similarly, at 5q12, the risk allele of a candidate causal variant had no effect on expression of predicted target genes *FGF10* and *MRPS30* (Ghoussaini et al., 2016).

An alternative method to study the effects of a (candidate causal variant in a) PRE is the Clustered Regularly Interspaced Short Palindromic Repeats (CRISPR)/CRISPR associated (Cas)9 gene editing system, which was first discovered in bacteria (Wiedenheft et al., 2012). Using CRISPR/Cas9 it has now become possible to, reliably and efficiently, introduce precise mutations in the human genome (Jinek et al., 2012). This gene editing technique makes use of a guide RNA (gRNA) that is complementary to the genomic region to be edited and a Cas9 enzyme that is guided by the gRNA to generate a double strand break (DSB) at this genomic region. The generated DSB can subsequently be repaired by either the non-homologous end joining pathway, which generally produces random insertions or deletions or by the homologous recombination repair pathway when a homology arm with the mutation of interest is co-transfected into the cells (Salsman and Dellaire, 2017). The latter pathway is able to generate specifically targeted mutations. At the 19p13.1 breast cancer locus this technique was used to generate a 57 base pair deletion containing the candidate causal SNP rs56069439. Lawrenson et al. showed a reduced *ANKLE1*, but not *ABHD8* or *BABAM1* expression as a result of this deletion (Lawrenson et al., 2016). A modified version of the Cas9 enzyme was used in the post-GWAS study by Betts et al. to silence PRE1 at 11q13, resulting in reduced *CUPID1, CUPID2* and *CCND1* expression (Betts et al., 2017). This nuclease-deficient Cas9 (dCas9) enzyme binds the target genomic region, but does not cleave the DNA. By fusion of dCas9 to various effector domains, CRISPR/Cas9 can be modified to a gene silencing or activation tool (Dominguez et al., 2016).

Interestingly, an average PRE has been predicted to regulate two or three different target genes (Sanyal et al., 2012). From the post-GWAS studies to date, evidence has now been presented for this at only 4 out of the 22 GWAS-identified breast cancer risk loci: 6q25.1, 10q21, 11q13, 19p13.1 (French et al., 2013; Darabi et al., 2015; Dunning et al., 2016; Lawrenson et al., 2016; Betts et al., 2017), which might suggest that maybe not all target genes have been identified yet at every locus investigated so far. Also considering the GWAS-identified breast cancer risk loci for which no post-GWAS analysis has been performed yet, there is still much work ahead.

Although the majority of the post-GWAS studies have followed this general pipeline for elucidating the functional mechanisms, one important step is still missing. Namely, evaluating of the tumorgenicity of the causal variants and the target genes in *in vitro* and *in-vivo* model systems, such as normal breast cancer cells or mice. Discovery of the genome-editing technique CRISPR/Cas9 has greatly enhanced our capabilities for taking this next step. Not only, because of the precision of this gene editing tool, but also because it allows for simultaneous genome-edits (Cho et al., 2013). However, there are certainly some challenges on this path and simply showing that the target gene is tumorigenic in an *in vitro* or *in vivo* model system is not sufficient, as it does not tie the germline variant to breast tumorgenicity. More subtle gene editing is necessary, and the question remains, whether this will always give a phenotype, since cancer risks conferred by these germline variants is low. This will probably be one of the biggest issues besides choosing the appropriate model system or animal.

## Discussion

In addition to the more than 170 GWAS-identified loci associated with breast cancer risk, 22 of these loci have been studied in more detail by post-GWAS analysis (Table 1). So far, the functional mechanism that candidate causal variants seem to make use of are mainly on the transcriptional level and deregulating target genes. In addition, the target genes involved do not seem to be specifically involved in DNA damage repair, like for high- and moderate-penetrant breast cancer risk genes, instead, somatic breast cancer drivers also appear to be enriched (Michailidou et al., 2017). Furthermore, the mechanisms that these causal variants use to confer breast cancer risk, are probably more complex than we anticipated, with often several iCHAVs at a GWAS-identified locus and some of them being able to regulate multiple target genes or ncRNAs (Table 1). Although we are not even half way this challenge, the availability of data on regulatory features, chromatin interactions and gene expression as well as the development of bioinformatics tools is definitely accelerating the process. However, in the future we could still benefit from more cistrome and interactome data on more TFs and on different cell types, especially normal breast cells. To facilitate more effective fine-scale mapping, more and larger case-control studies from African ancestry are necessary to benefit from the more structured LD in this population. Finally, we could also benefit from more paired genotype and gene expression data from normal breast samples for eQTL analysis as well as a variety of different normal breast epithelial cell-type models.

Regarding the GWAS-identified loci itself, it is obvious that more lower-risk variants predisposing to breast cancer risk still exist (Michailidou et al., 2017), however, again, larger sample sizes, especially for ER-negative breast cancer, as well as new statistical models to asses GWAS SNPs tagging causal variants with lower allele frequencies and smaller effect sizes are necessary (Fachal and Dunning, 2015). Interestingly, at the same time researchers are making use of alternative methods to identify novel breast cancer risk loci, which are mostly based on the same regulatory features that are also involved in exerting their biological function. Some of these features are gene expression, methylation and TF binding (Shenker et al., 2013; Xu et al., 2013; Anjum et al., 2014; Severi et al., 2014; van Veldhoven et al., 2015; Ambatipudi et al., 2017; Hoffman et al., 2017; Liu et al., 2017; Wu et al., 2018). In fact, the risk allele at 4q21 identified by Hamdi et al. was not discovered from GWAS, but from mapping SNPs associated with allele-specific gene expression in cancer-related pathway genes. The SNPs which were discovered in one dataset then act as proxies for allele specific expression and were evaluated for association with breast cancer risk in a second large GWAS study. Because the number of SNPs evaluated is reduced significantly as compared with GWAS, these type of analyses have more power and could thus identify lower risk alleles (Hamdi et al., 2016). These studies are called transcriptome-, epigenome- and phenome-wide association studies (TWAS, EWAS, and PheWAS) for gene expression features, methylation features and phenotypic features respectively. Interestingly, in the largest breast cancer TWAS to date, the expression levels of 48 genes were shown to be associated with breast cancer risk, of which 14 were novel and 34 were associated with known loci. However, 23 of these 34 genes were not previously identified as targets of GWAS-identified risk loci (Wu et al., 2018). This demonstrates that these types of studies are capable of identifying novel breast cancer risk loci, as well as validating previous GWAS-identified loci. EWASs, however, have not yet been very successful in identifying breast cancer risk loci associated with epigenetic changes, which is most likely a result of small sample sizes in these studies (Johansson and Flanagan, 2017). Finally, a recent PheWAS on multiple cancers, including breast cancer, has shown that using trait-specific PRS instead of single variants leads to improvement of the trait prediction power (Fritsche et al., 2018). In addition to these approaches, pathway-based analyses created to identify SNP-SNP interactions also open new avenues for identifying novel breast cancer risk SNPs and their interactors (Wang et al., 2017).

In this review, we have discussed the findings and lessons learned from post-GWAS analyses of 22 GWAS-identified risk loci. Identifying the true causal variants underlying breast cancer susceptibility provides better estimates of the explained familial relative risk thereby improving polygenetic risk scores (PRSs). Further stratification of their risk and contribution according the different subtypes of breast cancer and different populations will, however, be necessary. Moreover, unraveling the function of the causal variants involved in susceptibility to breast cancer increases our understanding of the biological mechanisms responsible for causing susceptibility to breast cancer, which will facilitate the identification of further breast cancer risk alleles and the development of preventive medicine for those women at risk for developing the disease.

## Author contributions

MR and AH designed the article and all authors wrote the article and approved of the final manuscript.

### Conflict of interest statement

The authors declare that the research was conducted in the absence of any commercial or financial relationships that could be construed as a potential conflict of interest.
